# The impact of emotions on the evaluation of environmentally damaging entrepreneurial opportunities: an experimental study

**DOI:** 10.3389/fpsyg.2026.1893664

**Published:** 2026-09-16

**Authors:** Marko Kolaković, Tin Horvatinović, Ivan Turčić

**Affiliations:** 1 Department of Entrepreneurship, Faculty of Economics and Business Zagreb, University of Zagreb, Zagreb, Croatia; 2 International Institute of Entrepreneurship, Samobor, Croatia

**Keywords:** affect, emotions, entrepreneurial opportunities, environmental harm, experiment

## Abstract

**Introduction:**

Prior scholarly work examined the effects of emotions on evaluations of entrepreneurial opportunities, which were typically non-context-specific. Thus, while the literature on the role of affect in judgments of entrepreneurial projects is prominent, the question of how eco-emotions are connected with evaluations of environmentally damaging entrepreneurial opportunities remains unanswered.

**Methods:**

This study utilized an experimental design to test the impact of three discrete emotions (hope, compassion, and anger) on the evaluation of environmentally damaging entrepreneurial opportunities, using a sample of 304 undergraduate students. The primary statistical methods employed were linear mixed-effects models and estimated marginal means contrasts.

**Results:**

In the screened sample, the anger condition was associated with statistically significant differences in more than one specification. Hope was context-dependent across opportunity types, whereas compassion did not differ significantly from the Control condition. Effect sizes were generally small; the two largest approached but did not reach the conventional medium benchmark. In the full-sample sensitivity analysis (N = 377), estimates were directionally similar, but the principal screened-sample findings were not confirmed.

**Discussion:**

These findings indicate that environmentally relevant emotions show different patterns in hypothetical entrepreneurial opportunity evaluation: anger showed the most consistent pattern, hope was context-dependent, and compassion showed no detectable difference relative to the Control condition. The findings contribute to the theoretical scope of the Appraisal Tendency Framework by examining emotion-specific patterns in the evaluation of environmentally damaging entrepreneurial opportunities, a judgment domain in which environmental harm is an explicit feature of the opportunity being evaluated. The variation observed across emotions and scenarios suggests potential boundary conditions for the application of the framework in this judgment domain. From a practical perspective, the findings suggest that emotional context may be relevant to understanding how environmentally damaging entrepreneurial opportunities are evaluated. However, given the student sample, generally small effect sizes, hypothetical scenarios, and sensitivity to the screening procedure, these practical implications should be considered preliminary and should not be interpreted as prescriptive guidance for entrepreneurs or policymakers.

## 1 Introduction

Entrepreneurial opportunity evaluation concerns judging a business idea or venture by its future potential for exploration or exploitation (Wood and McKelvie, 2015). Such judgments are not determined solely by the observable characteristics of an opportunity. They also depend on how individuals cognitively and affectively interpret uncertain future outcomes (Scheaf et al., 2020). Consequently, psychological processes have become increasingly important for understanding entrepreneurial opportunity evaluation (Frese and Gielnik, 2023).

Among these psychological processes, affect has received growing attention in entrepreneurship research. Discrete emotions have been shown to influence opportunity evaluation and related entrepreneurial judgments (Foo, 2011; Grichnik et al., 2010; Ivanova et al., 2018; Welpe et al., 2012). However, this literature has generally examined emotionally neutral entrepreneurial opportunities or emotions without a specific environmental context. This leaves open the question of whether emotions elicited by environmental concerns influence evaluations when environmental damage is itself an explicit characteristic of the entrepreneurial opportunity.

However, one common characteristic of many such studies is the lack of a specific context in which they were conducted, either on the affective or opportunity side, and often on both. Studies have often used emotion induction for non-specific purposes to test its effects on business cases with a neutral underlying business idea. This facet makes it difficult to make more specific, and consequently more methodologically sound, statements about the emotion-opportunity relationship. The environmental domain is one such example. Somewhat surprisingly, reviews of sustainable entrepreneurship noted considerable ambiguity regarding the effects of what could be labeled sustainable entrepreneurial activities on the environment (Muñoz and Cohen, 2018; Vedula et al., 2022), thereby calling for more diverse research within sustainable conceptual boundaries. Researchers have primarily focused on the benefits of sustainable entrepreneurship. Kuckertz and Wagner (2010) provided evidence that a sustainable orientation can drive individuals to become entrepreneurs, while Ploum et al. (2018) presented a strong case for pro-environmental values causally preceding opportunity recognition. Compared with non-psychological factors, psychological factors have been explored less often. One notable example is the work of Eller et al. (2020), who examined the role of entrepreneurial attitudes in shaping sustainable opportunity evaluations.

The psychological foundations of environmentally relevant judgments substantially predate the recent literature on eco-emotions. Foundational environmental psychology research has demonstrated the importance of differentiated environmental concerns and values (Schultz, 2001), psychological antecedents of environmentally significant behavior (Stern, 2000), and emotional affinity toward nature as a motivational basis for environmental protection (Kals et al., 1999). Recent research on discrete eco-emotions therefore builds on a longer tradition of examining psychological and affective antecedents of environmental judgment and behavior.

Far less is known about the role of psychological attributes in evaluations of environmentally damaging entrepreneurial opportunities. Here, the study of Qin et al. (2022) stands out. They discovered a strong direct effect of nature disengagement on the evaluation of environmentally damaging entrepreneurial opportunities. Nature disengagement, in their model, refers to cognitive coping mechanisms that enable environmentally harmful actions while minimizing self-impediments. This suggests that evaluation of environmentally damaging entrepreneurial opportunities is not solely a function of economic reasoning. In other words, a more complete understanding of how environmentally damaging entrepreneurial opportunities are evaluated requires consideration of underlying psychological processes. Referring back to the distinction between non-affect and affect research, the Qin et al. (2022) study falls into the former category. As for the latter, very little, if anything, is known about the role affect plays in evaluations of environmentally damaging entrepreneurial opportunities, which is unexpected given the cornucopia of published studies investigating the connection between discrete emotions and environmental concerns. Anger, hope, and compassion are among the most prominent and well-researched emotions in this context (Zaremba et al., 2024). For Zaremba et al. (2024), environmental anger is portrayed as outrage directed towards stakeholders with the power to take significant environmental actions, environmental hope reflects the belief that the current environmental trajectory can be altered for the benefit of the planet, and environmental compassion is an emotion stemming from experiencing harmful actions and, consequently, developing the tendency to rectify the situation. All three discrete emotions have been explored in an entrepreneurial context (Portocarrero et al., 2025), thereby providing conceptual and empirical grounding for examining their relevance to evaluations of environmentally damaging entrepreneurial opportunities.

Therefore, a research gap exists at the intersection of three prominent streams of research: opportunity evaluation, entrepreneurial affect, and sustainable entrepreneurship. As mentioned, the majority of publications on entrepreneurial emotions have examined general business opportunities rather than opportunities with explicit environmental harm. Similarly, environmental entrepreneurship research has focused on concepts such as pro-environmental intentions and sustainable opportunity recognition rather than on evaluating environmentally damaging entrepreneurial opportunities. As a result, there is a lack of empirical evidence on whether discrete emotions triggered by environmental content are relevant to the attractiveness of environmentally damaging entrepreneurial opportunities. This study addresses this gap by examining whether hope, compassion, and anger influence the evaluation of environmentally damaging entrepreneurial opportunities using an experimental design with a sample of undergraduate students.

Thus, this study aims to answer three research questions.

RQ1: Does hope influence the evaluation of environmentally damaging entrepreneurial opportunities?

RQ2: Does compassion influence the evaluation of environmentally damaging entrepreneurial opportunities?

RQ3: Does anger influence the evaluation of environmentally damaging entrepreneurial opportunities?

By answering these research questions, this study contributes to both entrepreneurial affect and environmental psychology. It examines whether emotion-specific patterns consistent with the Appraisal Tendency Framework extend to the evaluation of environmentally damaging entrepreneurial opportunities, in which environmental harm is an explicit characteristic of the opportunity being evaluated. The study does not propose a new appraisal dimension within the Appraisal Tendency Framework; instead, it examines emotion-specific patterns in this judgment domain and considers potential boundary conditions for applying the framework in this context. Environmentally damaging entrepreneurial opportunities therefore provide a setting in which environmental harm is an explicit feature of the opportunity being evaluated. This setting is also relevant to environmental psychology because it extends the study of eco-emotions beyond conventional outcomes such as environmental attitudes, behavioral intentions, and collective action to judgments concerning economic activities that generate negative environmental externalities. Accordingly, the experiment tests whether induced environmental emotions change evaluations of hypothetical environmentally damaging entrepreneurial opportunities, without implying effects on actual entrepreneurial behavior.

## 2 Methods

Using an experimental design to answer research questions in the domains of affective entrepreneurship (Portocarrero et al., 2025) and emotional responses to climate change (Zaremba et al., 2024) remains relatively uncommon, partly because standardized approaches to constructing targeted manipulations are limited. The availability of measures developed in the entrepreneurship (Qin et al., 2022) and emotion-related (Zaremba et al., 2024) literature makes such questions increasingly testable. Accordingly, an experiment was conducted in a classroom setting with university students to examine how emotional responses to environmental issues relate to business opportunity evaluations. The questionnaire was administered in accordance with the Faculty’s research procedures. The Ethics Committee of the Faculty of Economics & Business, University of Zagreb, approved the study.

The data were gathered using a one-session online questionnaire administered to students enrolled in an undergraduate entrepreneurship course in 2025 at the Faculty of Economics and Business, University of Zagreb, in the Republic of Croatia. Random assignment to the four experimental conditions was implemented through a physical draw. Copies of four unique QR codes, each linking to one of the four versions of the Google Forms questionnaire (Hope, Compassion, Anger, and Control), were printed on separate paper slips and placed together in an envelope with an equal number of slips for each condition. Each participant drew, without replacement, one paper slip and scanned the QR code with their phone, which directed them to the corresponding version of the questionnaire. Thus, survey software did not determine assignment to condition (Incekara-Hafalir et al., 2025). Before the draw, participants were not informed which QR code corresponded to which experimental condition. No separate allocation concealment procedure beyond the physical draw was used. Before beginning the questionnaire, students were briefly informed verbally about the study’s general purpose. The explanation was deliberately broad to minimize potential response bias while still providing sufficient information about the nature of the survey. Participants were not informed about the specific research questions or hypotheses before completing the questionnaire. The online questionnaire itself began with a concise description of the study’s purpose, followed by a consent question. After providing consent, participants read a set of stories about climate change, each designed to trigger certain emotions. There were four sets of stories: one to stimulate hope, one to stimulate compassion, one to stimulate anger, and one as a control. These four sets of stories defined the four experimental conditions. In other words, the only difference among the four survey groups was the set of environmental stories presented. Each experimental condition comprised seven short stories. In the English-language versions reproduced in the Supplementary material, individual stories ranged from 30 to 73 words (hope: 45–72; compassion: 37–73; anger: 30–58; control: 46–66). Within each condition, the seven stories were presented in the same fixed order to all participants. The complete English-language wording of the stories is provided in the Supplementary material; the materials administered to participants were translated into Croatian as described below. The time spent reading the emotion-induction stories was not recorded separately. After reading their assigned stories, the participants were asked a straightforward question about the stories they read, with one correct answer out of three provided options. This question served as the first step in identifying unengaged respondents. Next, the participants rated their emotions of hope, compassion, anger, guilt, and anxiety, regardless of their group. These ratings provided the manipulation check for the targeted emotions. Following emotional ratings, the participants read two case studies describing environmentally damaging entrepreneurial opportunities. After each project description, participants answered a straightforward question about the project, with one correct answer out of three provided options. As with the question about the emotion-induction stories, these two questions served as safeguards for identifying unengaged respondents. For each case study, participants answered questions assessing how they evaluate the project’s business prospects. Next came a neutral case study. The neutral case study was placed last to distract participants before they answered eight questions about their general stances on potentially damaging environmental actions. In other words, an environmentally neutral project mitigates the potential bias in general environmental opinions. Lastly, the participants were asked about their age, gender, previous work experience, and family entrepreneurial background.

Overall, 377 students completed the survey. However, not all survey responses were included in the final analysis. As mentioned, there were three questions (one after a set of stories, one after Case One, and one after Case Two) designed to uncover unengaged respondents. Therefore, if a respondent provided an incorrect answer to one or more of the three questions, they were flagged as unengaged and excluded from the sample. Furthermore, the variability in ratings of emotions, project evaluations, and stances on environmental actions was assessed using the standard deviation metric. Accordingly, if a respondent showed no response variability in one or more of the three question categories, they were labeled as unengaged and removed from the final analysis. Applying these screening criteria reduced the final sample size to 304. All 304 survey responses contained no missing values due to the questionnaire design. Figure 1 shows the entire process of obtaining the final sample as a CONSORT diagram.

**Figure 1:**
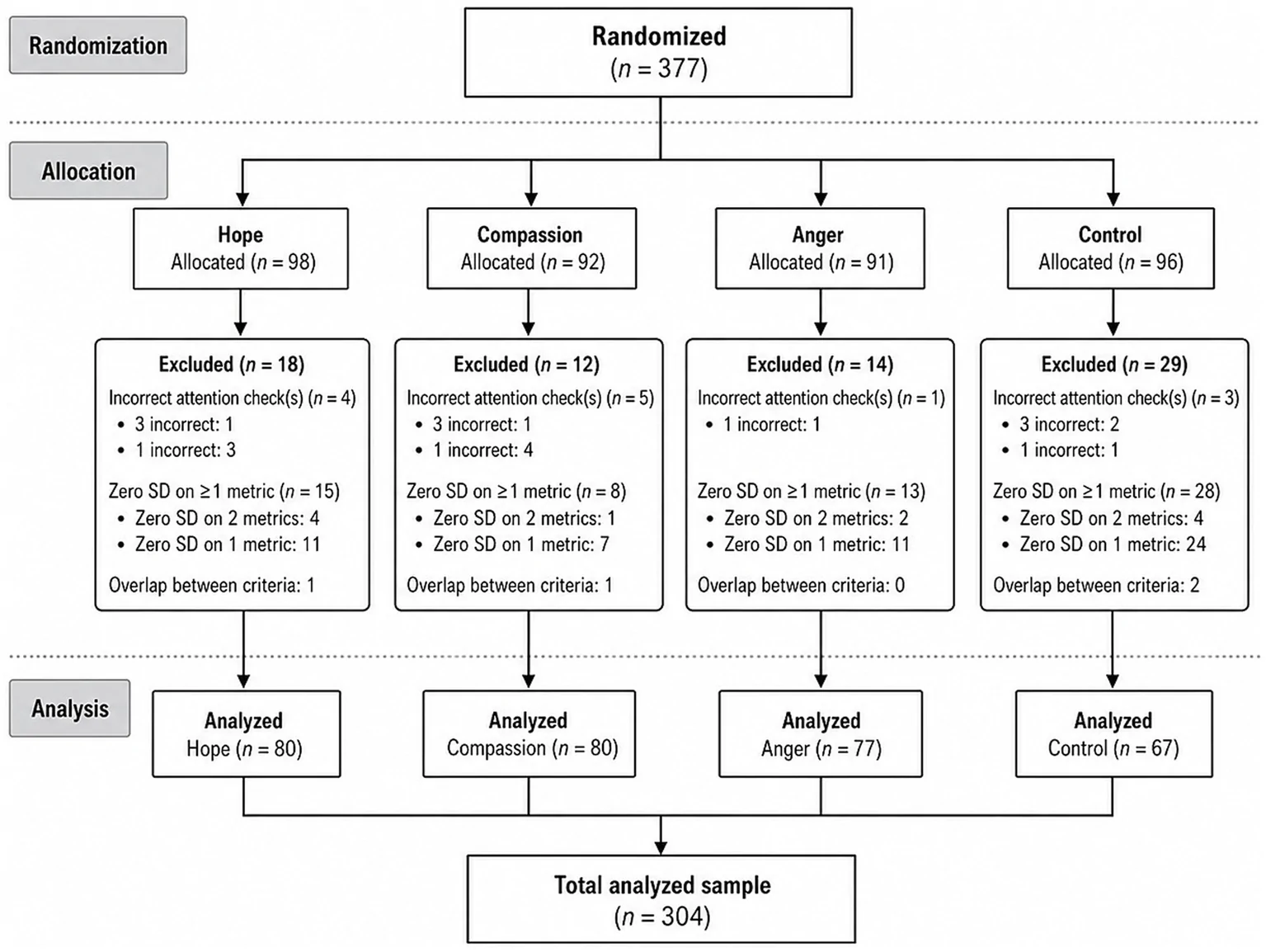
CONSORT diagram for sample composition.

For the survey data, this research used text from previous publications to construct the variables. All survey items are provided in the Supplementary material.

This study used the dataset of short stories published by Zaremba et al. (2024) to induce emotions. Zaremba et al. (2024) compiled short stories designed to trigger certain emotions. These authors report that reliable stories aim to manipulate hope, compassion, and anger. The same cannot be said of guilt and anxiety since, in those cases, there is overlap in ratings of emotional levels with others. For this reason, the authors of this paper included guilt and anxiety only as a way of testing the realization of planned emotions. While there is no deterministic formula for determining how many stories to include, Aguinis and Bradley (2014) recommended using at least five longer scenarios for each manipulation target. The authors of this paper exercised research discretion and ultimately selected seven stories due to their brevity. Once the number of stories was decided on, the authors examined the ranking of stories for each emotion, as provided by Zaremba et al. (2024). These rankings were not the sole determinant of inclusion. Each story has a topic that can overlap with the business opportunities (described next). Therefore, the authors of this paper selected the thirteen most highly rated stories for each emotion (hope, compassion, and anger) and, through discussion, selected seven stories whose topics are not connected to the business projects.

*Environmentally damaging entrepreneurial opportunities* are drawn from Qin et al. (2022), who devised three nature-harming scenarios. The business opportunities focus on air pollution, water pollution, and animal cruelty. For this study, the animal cruelty case was not presented to the participants for two reasons. First, since robustness was one of the aims of including multiple scenarios, the air and water pollution case combination is the closest in resemblance to the other combinations that include animal cruelty. Second, adding three longer case studies to an already expansive questionnaire could produce participant fatigue, in turn reducing response quality. Following Keh et al. (2002), as did Qin et al. (2022), participants rated business opportunities using three questions on a seven-point Likert scale. To address an environmentally neutral business opportunity, an often-cited case study by Keh et al. (2002) is adapted.

In addition to the main variables of interest, the survey included measures to control for the postulated effects. The first control variable, *nature disengagement* (Qin et al., 2022), is composed of eight questions rated on a seven-point Likert scale. Nature disengagement reflects individuals’ moral justifications for nature-damaging actions in a business context. Given the theoretical development elaborated in Qin et al. (2022), nature disengagement is likely a stable trait that influences decisions to harm nature. Thus, it is accounted for here as a potential predictor of environmentally damaging entrepreneurial opportunities.

Standard demographic controls commonly used in entrepreneurship research were also included. The participants answered demographic questions about their *gender* (coded: 0 = female, 1 = male) and *age*. They also stated whether they had *prior work experience* (coded: 0 = no previous work experience, 1 = previous work experience) and whether they had a *family member who is an entrepreneur* (coded: 0 = no entrepreneur in the family, 1 = entrepreneur in the family).

The source materials used to construct these variables were originally published in English and were therefore translated into Croatian. All authors of this paper were involved in the translation process, and the text was deemed acceptable once all authors approved it. However, due to the possibility of blind spots, before it was administered to students, the survey was completed by three people (one student from another faculty and two non-student working individuals) to pinpoint potential translation and wording issues. Their suggestions were considered by all authors and applied if all three agreed they improved the text. It is worth noting that the text was kept gender-neutral to avoid this kind of bias. More specifically, gender names were altered across all story sets to achieve equal representation. In the project evaluations, the authors used a single letter to identify individuals rather than their full names. This practice was adopted due to the specifics of the Croatian language. Otherwise, the whole text needs to be adapted, leading to confusion for the reader.

The numerical analysis of the gathered data proceeded in several stages, similar to those of recently published studies that used a similar research design to address discrepant research questions (e.g., Dew et al., 2024; Forbes et al., 2024; Snytte et al., 2025; Tang et al., 2023). The preliminary analysis consisted of five steps. First, commonly used descriptive statistics of the sample (mean and standard deviation) are reported. Second, the success of randomization is tested using Welch ANOVAs for non-binary and Pearson’s χ^2^ tests for binary control variables. Third, a series of t-tests is conducted to assess the effectiveness of inducing emotions. Independent-sample Welch t-tests determine between-group emotion inducement and nature disengagement stability, while paired-sample t-tests determine within-group emotion inducement. Fourth, the internal consistency of the two opportunity evaluation scales and the nature disengagement scale is assessed using Cronbach’s alpha coefficient. Fifth, a standard correlation matrix is presented.

Next, a repeated-measures linear mixed-effects model fitted under the restricted maximum likelihood (REML) criterion is used to assess the effects of the chosen variables on opportunity evaluations. Each participant contributed one observation to the Air case and one observation to the Water case. The model fixed effects are Group, Case, Group-Case interaction, Disengagement, Gender, Age, Work, and Family. Because of the experimental design, the fixed-effects composition was selected *a priori*, meaning no data-driven selection procedure was used. Given the non-independence of participant evaluations, the model includes a participant-specific random intercept. A random-intercept specification is preferred over a random-slope specification because a random-slope specification includes more covariance parameters than can be separately identified from the within-participant covariance structure in a model with two observations per participant. The type III ANOVA and regression coefficient t-tests use the Satterthwaite denominator degrees of freedom. For the type III ANOVA calculations, partial η^2^ metrics are included as measures of effect size, as additional information to the standard F-statistics. For the regression model, standard regression coefficient estimates are supplemented with parametric bootstrap 95% percentile confidence intervals based on 10,000 samples. Furthermore, the participant random-intercept variance and residual variance are extracted from the fitted model. An unconditional intraclass correlation coefficient (ICC) is calculated from an intercept-only model, while an adjusted ICC is calculated from the final model. Marginal R^2^ and conditional R^2^ values are calculated to quantify the variance explained by the fixed effects and by the complete mixed-effects model, respectively.

The final set of model calculations pertains to estimated marginal means contrasts for Group overall and separately for the Air and Water cases, totaling nine contrasts. Contrasts in these models are specified as Dunnett-type contrasts since the comparisons included more than one treatment group against the same control group. Degrees of freedom are calculated using the Kenward-Roger method. Overall marginal means are averaged across the two case studies, categorical control variables are averaged across factor levels, while continuous covariates are set to their reference-grid means. Multivariate-t (MVT) adjusted *p*-values are reported to account for family-wise error, while global Holm-adjusted *p*-values are produced to obtain more stringent significance inferences. Additionally, Cohen’s d values are calculated for each of the nine contrasts to interpret the effect sizes. For all nine contrasts, a family-wise bootstrap Bonferroni-adjusted procedure on 10,000 samples is performed to obtain 95% confidence intervals.

The assumptions of the linear mixed-effects model are evaluated using both graphical and numerical diagnostics since some formal tests are sensitive to normality deviations. Specifically, the plot of residuals versus fitted values is used to infer homoscedasticity (if no funnel-type patterns are present) and linearity (if no curvilinear patterns are present). Two normal Q-Q plots provide one point for assessing the normality assumption for participant-level deviations and residuals when points are distributed along their mapped straight lines. These visual assessments are supplemented by numerical summaries of the scaled residuals, Shapiro–Wilk tests, skewness, and excess kurtosis for both the scaled residuals and participant random intercepts. In addition, variance inflation factors are calculated to rule out multicollinearity. Also, the fitted linear mixed-effects model is checked for singularity and convergence.

Lastly, two approaches are implemented to evaluate the robustness of the main findings. First, potentially influential participants are detected using Cook’s distances with a 4/N threshold, where N is the sample size. The removal of identified influential participants is used solely for sensitivity analysis of the estimated marginal means. Second, the entire numerical methodological procedure described in this section is carried out on the full sample of 377 students, with no participant removal.

The abbreviations in the following Results section are: Hope (induced hope), Compassion (induced compassion), Anger (induced anger), Air (air pollution business opportunity), Water (water pollution business opportunity), Disengagement (measure of nature disengagement), Age (age of the participant), Work (previous work experience), Gender (gender of the participant), and Family (entrepreneurial background in the family of the participant).

## 3 Results

Table 1 shows detailed descriptive statistics of the entire sample. At the study level, around 64% of participants were female, around 85% had previous work experience, and around 45% had an entrepreneur in their family. The average age of the participants is around 21. Similar numbers are found when participants are divided into Hope, Compassion, Anger, and Control groups.

**Table 1:** Descriptive statistics.

Variable		Mean	SD
Overall *N* = 304	Air	3.855	1.361
	Water	4.218	1.583
	Disengagement	2.313	0.780
	Age	21.454	1.571
	Work	0.852	-
	Gender	0.359	-
	Family	0.451	-
Hope *N* = 80	Air	4.017	1.475
	Water	3.996	1.519
	Disengagement	2.292	0.793
	Age	21.625	2.645
	Work	0.850	-
	Gender	0.425	-
	Family	0.438	-
Compassion *N* = 80	Air	3.850	1.419
	Water	4.313	1.518
	Disengagement	2.302	0.842
	Age	21.263	0.759
	Work	0.900	-
	Gender	0.363	-
	Family	0.513	-
Anger *N* = 77	Air	3.481	1.207
	Water	3.935	1.614
	Disengagement	2.286	0.637
	Age	21.532	1.021
	Work	0.779	-
	Gender	0.286	-
	Family	0.390	-
Control *N* = 67	Air	4.100	1.247
	Water	4.697	1.607
	Disengagement	2.381	0.848
	Age	21.388	0.984
	Work	0.881	-
	Gender	0.358	-
	Family	0.463	-

Formal testing of the similarities noted across design groups provides a basis for assessing the success of randomization. Such results are presented in Table 2. According to Table 2, neither the Welch ANOVA nor Pearson’s χ^2^ test is significant, indicating no differences between groups for Age, Disengagement, Gender, Work, and Family. Therefore, the randomization process was correctly executed.

**Table 2:** Randomization checks.

Variable	Welch ANOVA	Pearson’s χ^2^
Age	1.427 (3, 159.16)	-
Disengagement	0.205 (3, 163.34)	-
Gender	-	3.318 (3)
Work	-	5.133 (3)
Family	-	2.490 (3)

Degrees of freedom in brackets.

Next, a series of t-tests is performed within and between groups to assess the extent to which the manipulations were effective. The exact values of these tests are presented in Table 3. When examining the core manipulations of emotions, there are significant differences between the three manipulation groups (Hope, Compassion, and Anger) and the Control group. Also, within each group, the targeted emotion (Hope, Compassion, and Anger) is larger than all the other four measured emotions. Interpreted jointly, these results demonstrate that only targeted emotions were triggered, and they were more pronounced than in the Control group. In addition, there were no significant differences across all four groups in Disengagement, indicating that Disengagement remained a stable trait. All of the results in Table 3 lend high confidence in obtaining the desired data structure for formally testing the effects of emotions on project evaluations.

**Table 3:** Manipulation checks.

Variable	Control	Hope	Compassion	Anger	Guilt	Anxiety
Emotions						
Hope	9.238^***^ (127.94)	-	6.344^***^ (79)	18.018^***^ (79)	15.188^***^ (79)	16.248^***^ (79)
Compassion	4.190^***^ (140.52)	9.138^***^ (79)	-	6.744^***^ (79)	14.099^***^ (79)	5.368^***^ (79)
Anger	12.121^***^ (139.46)	12.245^***^ (76)	11.353^***^ (76)	-	8.219^***^ (76)	4.501^***^ (76)
Disengagement						
Hope	−0.648 (136.77)	-	−0.073 (157.45)	0.056 (150.20)	-	-
Compassion	−0.565 (140.11)	-	-	0.133 (146.89)	-	-
Anger	−0.750 (121.24)	-	-	-	-	-

^***^ Means significant at 1% level.

Degrees of freedom in brackets.

The variables in the first column are referenced ones.

Table 4 presents the correlation matrix and internal consistency measures for the latent variables. All three latent constructs (Air, Water, and Disengagement) have Cronbach’s alphas above the recommended cut-off of 0.7, indicating consistent measurement of their respective survey items. Of the variables shown in Table 4, significant correlations are found for all three combinations of Air, Water, and Disengagement, as well as for the significant correlation between Disengagement and Gender.

**Table 4:** Internal consistency and correlation matrix.

Variable	α	Air	Water	Disengagement	Age	Work	Gender	Family
Air	0.856	-						
Water	0.890	0.475^***^	-					
Disengagement	0.705	0.280^***^	0.335^***^	-				
Age	-	−0.008	−0.016	−0.033	-			
Work	-	0.140^**^	0.052	−0.086	0.020	-		
Gender	-	0.076	0.053	0.220^***^	−0.011	0.003	-	
Family	-	−0.033	0.009	−0.007	−0.014	−0.051	−0.084	-

^***^ Means significant at 1% level, ^**^ means significant at 5% level.

α means Cronbach’s alpha.

Table 5 displays the analysis of variance of the main model. The principal significant value of interest is the Group statistic. Therefore, at least one emotion significantly differs from the control when Air and Water are combined. Regarding other significant factors, the participants rated the two types of case studies differently overall and marginally between groups. Also, Disengagement and Work were associated with business opportunity evaluations. Regarding effect sizes for the significant statistics, Disengagement is only of medium strength; all others fall into the small-strength category.

**Table 5:** Analysis of variance.

Effect	F-statistic	Effect sizes
Group	3.090^**^ (3, 295)	0.030
Case	18.481^***^ (1, 300)	0.058
Disengagement	45.388^***^ (1, 295)	0.133
Gender	0.089 (1, 295)	0.000
Age	0.001 (1, 295)	0.000
Work	5.087^**^ (1, 295)	0.017
Family	0.073 (1, 295)	0.000
Group * Case	2.470^*^ (3, 300)	0.024

^***^ Means significant at 1% level, ^**^ means significant at 5% level, ^*^ means significant at 10% level.

Degrees of freedom in brackets.

Group has 4 categories (Hope, Compassion, Anger, and Control as reference); Case has 2 categories (Water and Air as reference).

Next, Table 6 exhibits the values of the linear mixed-effects model estimates, which are consistent with the bootstrap confidence intervals. For the three emotion conditions, Anger had a significant negative coefficient for Air relative to the Control condition (95% CI: −0.982, −0.060). The Anger × Water interaction was negative but non-significant (95% CI: −0.638, 0.361). The Compassion coefficient for Air (95% CI: −0.658, 0.241) and the Compassion × Water interaction coefficient (95% CI: −0.618, 0.358) were both not significant. Hope did not differ significantly from Control for Air (95% CI: −0.472, 0.448), whereas the Hope × Water interaction was significantly negative (95% CI: −1.111, −0.118), indicating that the effect of Hope differed between the two case studies and was more negative for Water. Other variables associated with variability in business evaluations included Disengagement (95% CI: 0.420, 0.767), Work (95% CI: 0.057, 0.799), and the type of case study (95% CI: 0.222, 0.958). The participant random-intercept variance is 0.786 (SD = 0.887), while the residual variance is 1.140 (SD = 1.068).

**Table 6:** Linear mixed model regression.

Predictor	Estimates	95% Bootstrap CI
Hope	−0.015 (0.231)	[−0.472, 0.448]
Compassion	−0.209 (0.230)	[−0.658, 0.241]
Anger	−0.525^**^ (0.233)	[−0.982, −0.060]
Disengagement	0.596^***^ (0.088)	[0.420, 0.767]
Gender	−0.043 (0.144)	[−0.319, 0.240]
Age	0.001 (0.043)	[−0.083, 0.084]
Work	0.431^**^ (0.191)	[0.057, 0.799]
Family	−0.037 (0.136)	[−0.299, 0.236]
Water	0.597^***^ (0.184)	[0.222, 0.958]
Hope * Water	−0.618^**^ (0.250)	[−1.111, −0.118]
Compassion * Water	−0.134 (0.250)	[−0.618, 0.358]
Anger * Water	−0.142 (0.252)	[−0.638, 0.361]

^***^ Means significant at 1% level, ^**^ means significant at 5% level.

Standard errors in brackets.

Conditional R^2^ is 0.494; marginal R^2^ is 0.145.

The required assumptions of the linear mixed-effects model were evaluated through several diagnostic checks. First, the plot in Figure 2 shows no signs of heteroscedasticity or nonlinearity, as there are no funnel or curvilinear patterns. Second, the Q-Q plots in Figures 3, 4 closely follow the reference line, with slight deviations in the tails, indicating approximate normality of residuals and participant-level deviations (random intercepts), respectively. The scaled conditional residuals ranged from −2.27 to 2.67. Only 5 of 608 residuals exceeded an absolute value of 2, and none exceeded an absolute value of 3. However, Shapiro–Wilk tests indicated non-normality for both scaled residuals (W = 0.993, *p* = 0.010) and participant random intercepts (W = 0.988, *p* = 0.012). Nevertheless, the magnitudes of the distributional deviations are modest since residual skewness is −0.147 with excess kurtosis of −0.308, while random-intercept skewness is −0.165 with excess kurtosis of −0.595. Taken together, the diagnostic evidence indicates no substantial departures likely to compromise the mixed-model inference. Third, there are no multicollinearity issues, as the VIF values for all non-interaction variables are below 5. Fourth, the fitted linear mixed-effects model is not singular and converged successfully. Fifth, the unconditional ICC is 0.448, and the ICC of the final model is 0.408, supporting the use of a mixed-effects specification. Thus, the diagnostics indicated no meaningful violations likely to compromise mixed-model inferences.

**Figure 2:**
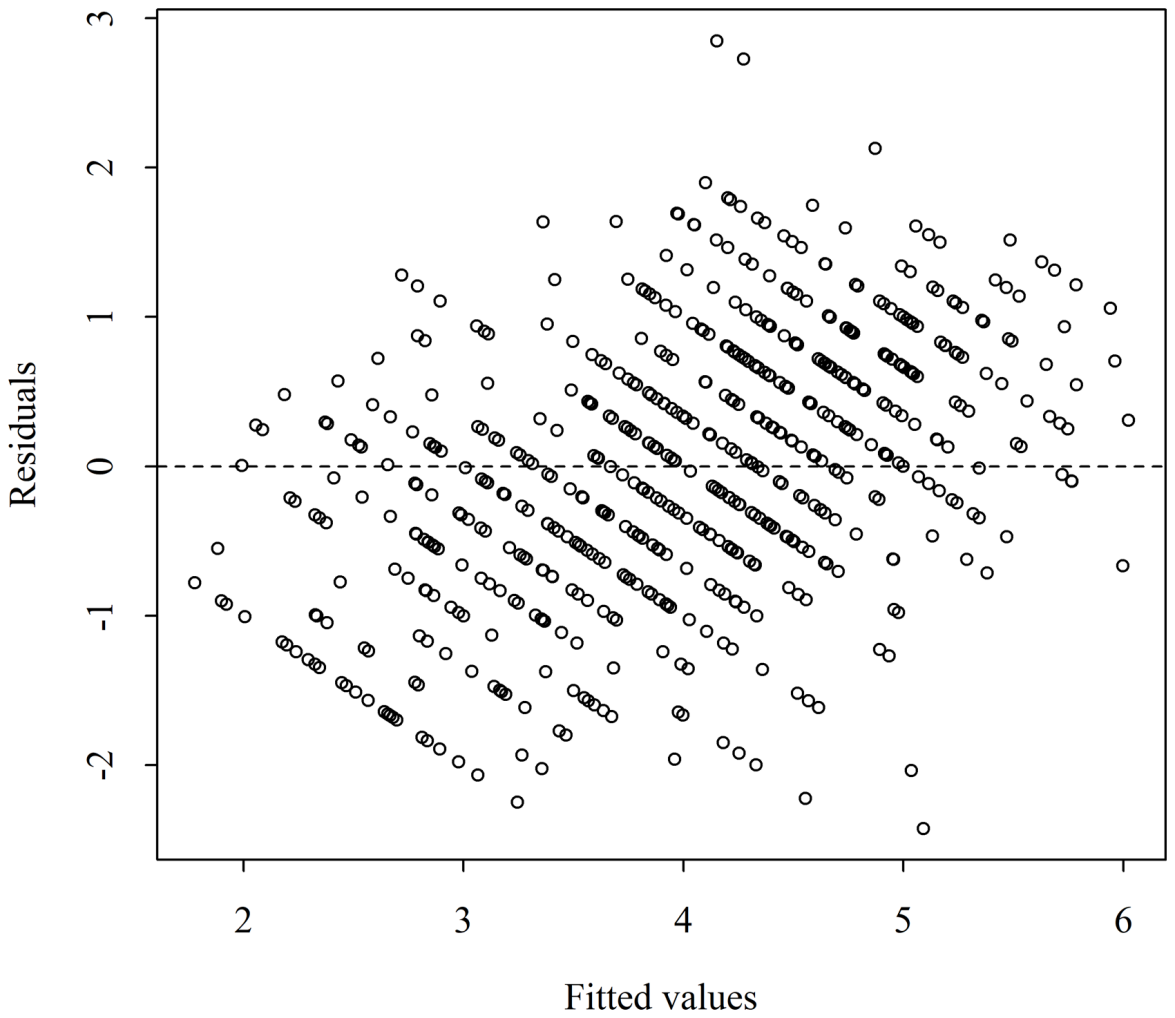
Residuals versus fitted values.

**Figure 3:**
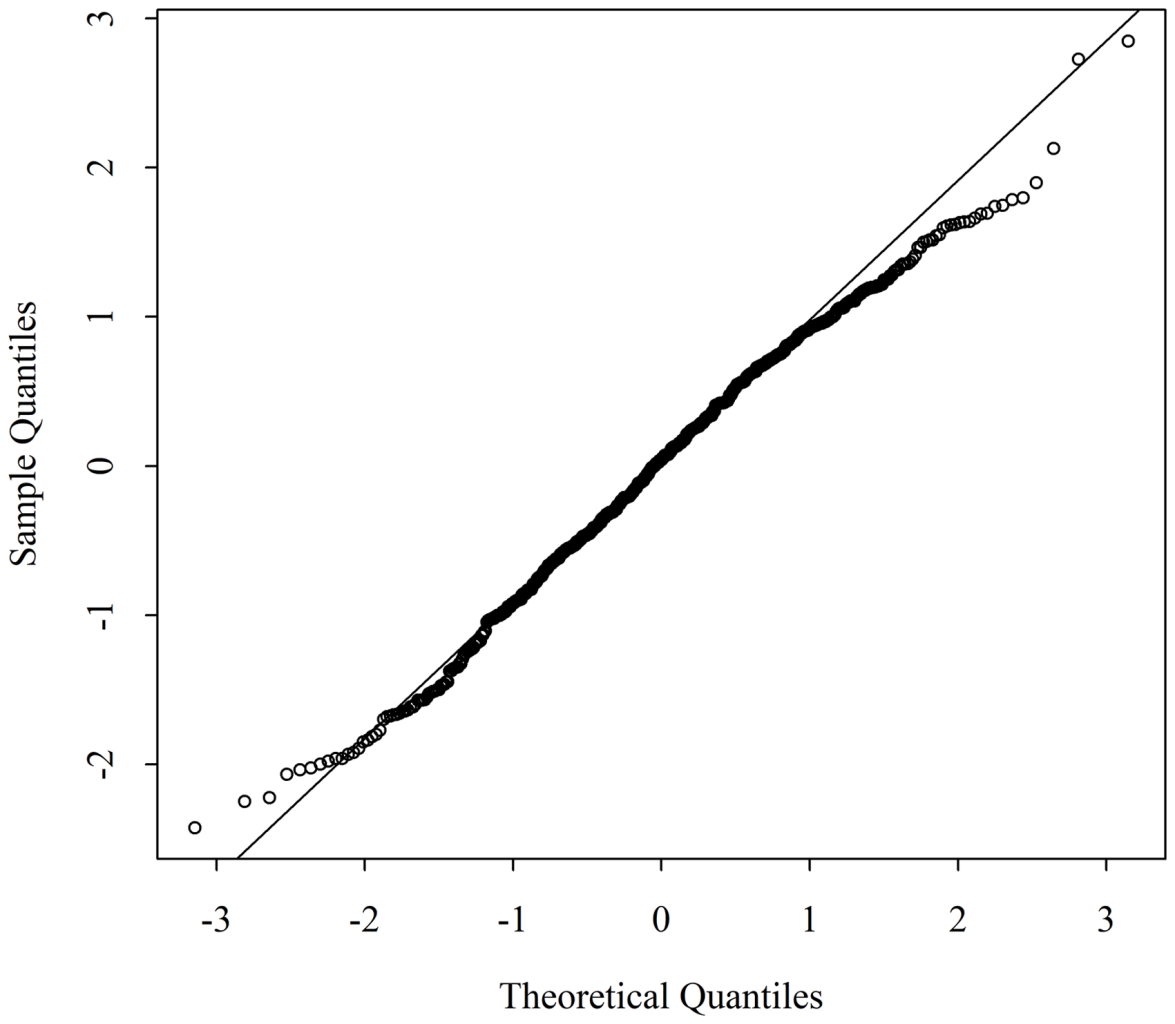
Q–Q plot of residuals.

**Figure 4:**
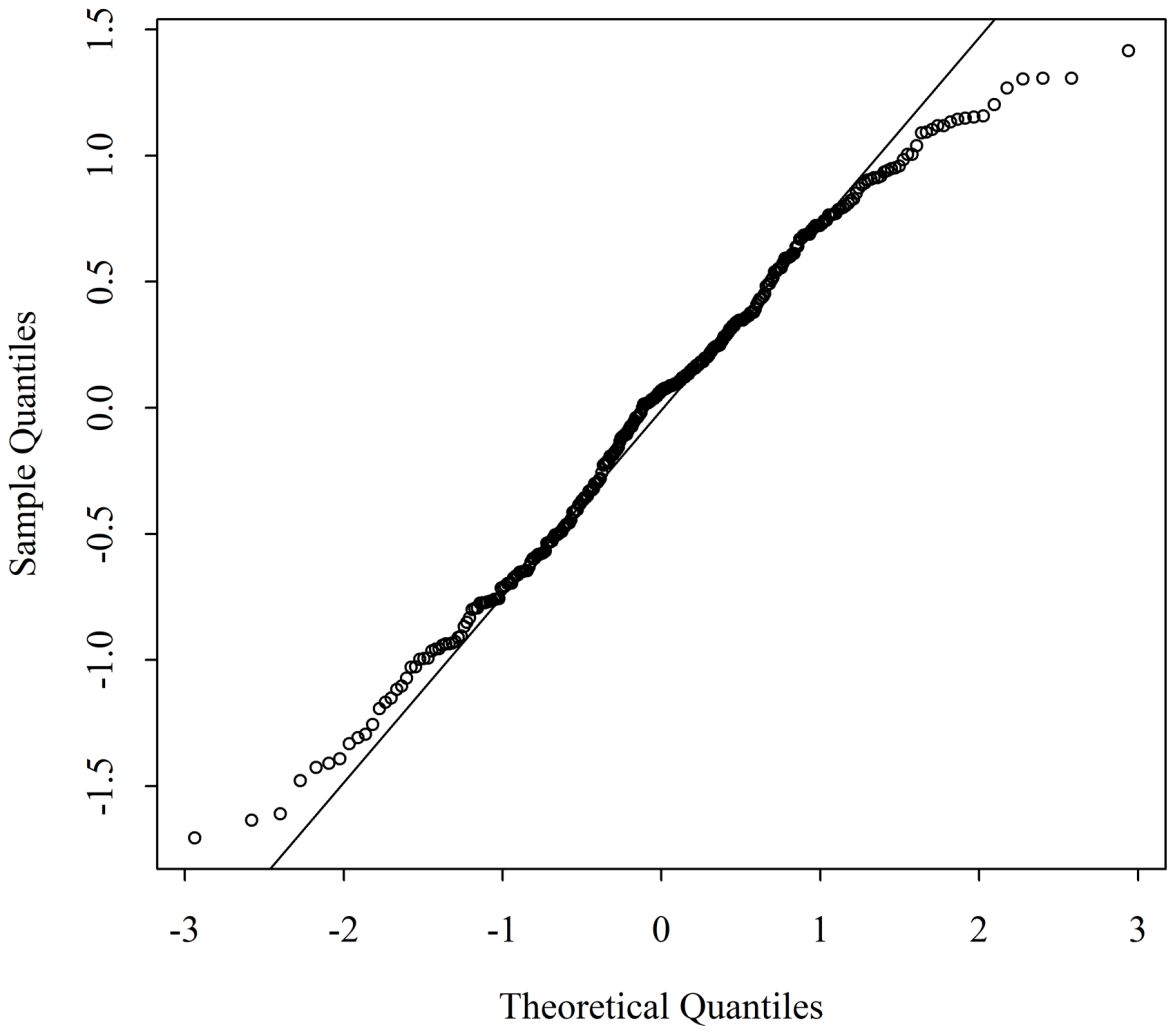
Q–Q plot of random intercepts.

Lastly, Table 7 reports marginal means contrasts of the combined case types and their individual estimates. Anger is the only emotion with almost all significant estimates in all three specifications (Overall: [95% FW CI: −1.138, −0.043]; Air: [95% FW CI: −1.144, 0.122]; Water: [95% FW CI: −1.309, −0.006]) for both adjusted *p*-value estimates, all of which are negative. One estimate, that for Air, is marginal (MVT adjusted *p*-value) and non-significant (Global Holm *p*-value). Compassion has no significant estimates in any of the three specifications (Overall: [95% FW CI: −0.795, 0.256]; Air: [95% FW CI: −0.841, 0.421]; Water: [95% FW CI: −0.970, 0.286]). Hope is significant only in the Water specification, with a negative coefficient (95% FW CI: −1.258, −0.003). In other specifications, combined case studies (95% FW CI: −0.863, 0.199) and Air (95% FW CI: −0.641, 0.600), Hope is not significant. Moreover, the bootstrap family-wise 95% confidence intervals confirm all the initial contrast estimates inferences based on Global Holm *p*-values from Table 7. Using Cohen's (1988) conventional benchmarks for standardized mean differences (d = 0.20 small, d = 0.50 medium, and d = 0.80 large), most estimated contrasts were small. The two largest estimates, Anger (d = 0.481) and Hope (d = 0.456) in the Water case, approached but did not reach the conventional medium benchmark. The Anger contrasts for Overall (d = 0.429) and the Air case (d = 0.378) likewise fell between the conventional small and medium benchmarks. These thresholds are general conventions rather than domain-specific standards of practical importance, so the effect sizes are interpreted cautiously.

**Table 7:** Estimated marginal means contrasts.

	Contrast	Estimates	Cohen’s d	MVT-adjusted *p*-value	Global Holm p-value	95% family-wise bootstrap CI
Overall	Hope – Control	−0.324 (0.194)	0.234	0.221	0.476	[−0.863, 0.199]
	Compassion – Control	−0.276 (0.193)	0.199	0.340	0.546	[−0.795, 0.256]
	Anger – Control	−0.596^***^ (0.196)	0.429	0.007	0.024	[−1.138, −0.043]
Air	Hope – Control	−0.015 (0.231)	0.011	1	0.947	[−0.641, 0.600]
	Compassion – Control	−0.209 (0.230)	0.150	0.681	0.730	[−0.841, 0.421]
	Anger – Control	−0.525^*^ (0.233)	0.378	0.064	0.150	[−1.144, 0.122]
Water	Hope – Control	−0.633^**^ (0.231)	0.456	0.017	0.044	[−1.258, −0.003]
	Compassion – Control	−0.343 (0.230)	0.247	0.305	0.546	[−0.970, 0.286]
	Anger – Control	−0.667^**^ (0.233)	0.481	0.012	0.035	[−1.309, −0.006]

Cohen’s d values are in absolute terms.

*p*-values indicated by stars are values without multiplicity adjustments; ^***^ means significant at 1% level, ^**^ means significant at 5% level, ^*^ means significant at 10% level.

Standard errors in brackets.

Moreover, the statistically significant estimated marginal means contrasts remain as such after a re-analysis on a sample where five influential participants, as identified by Cook’s distance, are excluded.

Figure 5 shows the estimated marginal means and their 95% model-based (non-adjusted) bootstrap confidence intervals across the experimental conditions.

**Figure 5:**
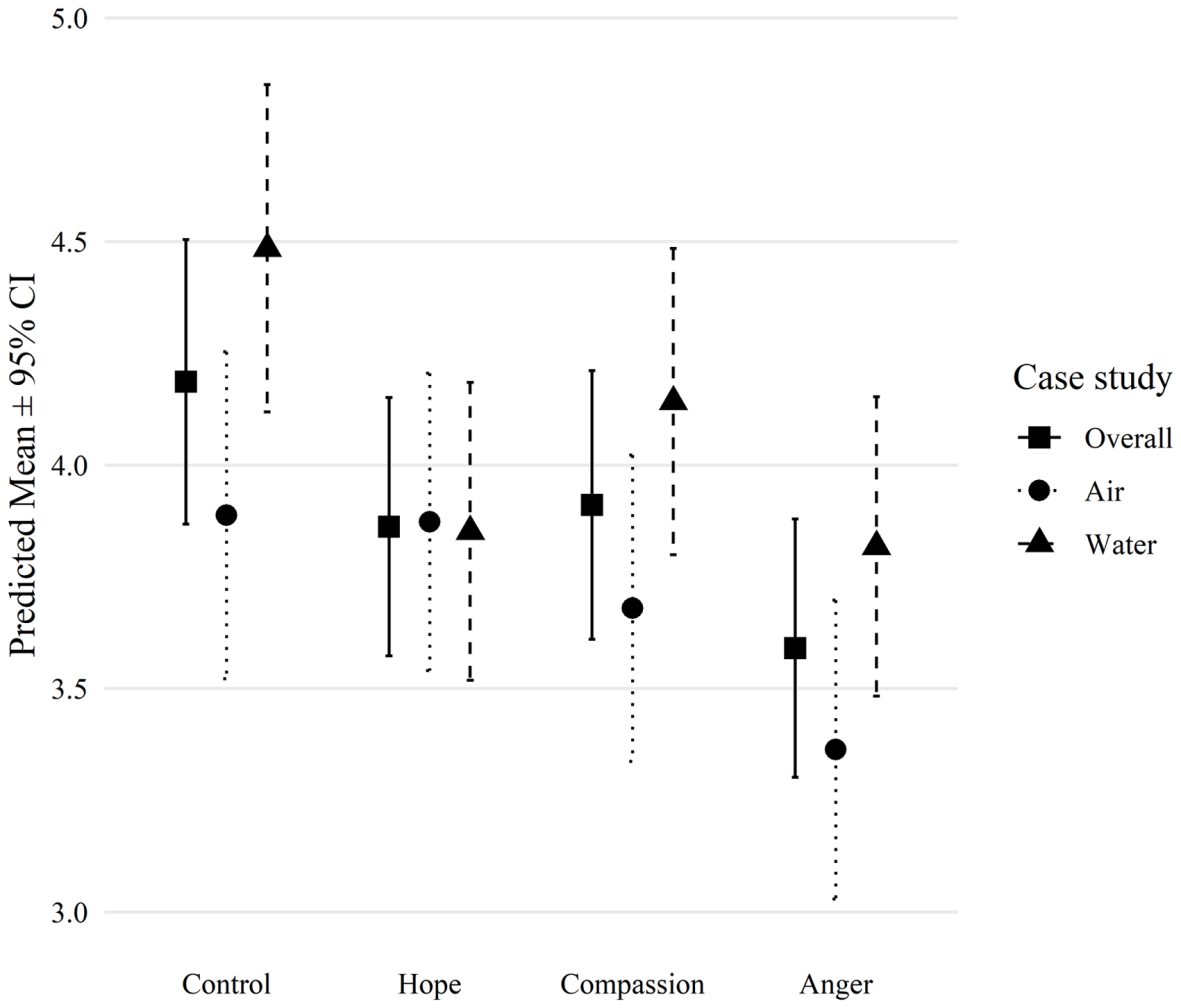
Estimated marginal means and 95% model-based bootstrap confidence intervals.

Finally, the robustness of the results is checked using the full sample of undergraduate students (*N* = 377). The findings from the full sample portray a similar picture to the final sample in terms of directionality, but not in terms of interpretation. Although the full-sample output for randomization and emotion elicitation was replicated, the findings from the linear mixed-effects model and estimated marginal means contrasts were not. Of the statistically significant estimates in the linear mixed-effects model, CaseStudy and Disengagement remained significant in the Type III analysis, and the Hope × Water interaction remained significant in the regression analysis, while others did not pass the standard threshold of significance. Likewise, for the estimated marginal means contrasts, the significant estimates remained significant only before multiplicity adjustments, meaning they were non-significant when MVT-adjusted *p*-values and Global Holm *p*-values were used as the benchmark.

## 4 Discussion

Taken together, the findings indicate that the three environmentally relevant emotions do not operate interchangeably when individuals evaluate environmentally damaging entrepreneurial opportunities. The anger condition showed the most consistent pattern of lower evaluations across the evaluated scenarios, whereas the pattern for hope was context-dependent and the compassion condition did not differ significantly from the Control condition in opportunity evaluation. The theoretical importance of this pattern lies in the emotion-specific nature of the judgments, consistent with the central premise of the Appraisal Tendency Framework that discrete emotions influence judgment through distinct appraisal tendencies rather than through positive or negative valence alone.

However, the principal emotion-related findings did not remain statistically significant in the full-sample sensitivity analysis (*N* = 377), which limits the robustness of the screened-sample results. The screened sample is retained as the primary inferential target because the study’s engagement criteria were designed to reduce measurement error associated with inattentive responding (Oppenheimer et al., 2009; Maniaci and Rogge, 2014). Nevertheless, exclusion based on attention checks may introduce selection bias (Montgomery et al., 2018; Mathur, 2025), so the central findings should be interpreted as conditional on the screening procedure and in need of replication.

### 4.1 Interpreting the findings

Following previous entrepreneurship studies examining emotions in opportunity assessments (e.g., Foo, 2011; Ivanova et al., 2018), we interpret the findings using the Appraisal Tendency Framework (Lerner and Keltner, 2000). The Appraisal Tendency Framework posits a specific configuration of appraisal tendencies for each emotion, in addition to its valence, to inform judgments or behavioral actions. The cognitive predisposition to interpret a future event begins with the activation of an emotion and extends beyond it (Lerner et al., 2015). Thus, emotions shape the cognitive interpretations by which individuals evaluate events in their surroundings. In this study, these are environmentally damaging entrepreneurial opportunities.

Anger is associated with appraisal tendencies including high certainty, high individual control, and greater attribution of responsibility to others (Lerner et al., 2015; Lerner and Keltner, 2000; Smith and Ellsworth, 1985). These tendencies can make events appear more understandable and controllable and can increase the attribution of blame to specific individuals or groups (Lerner et al., 2015; Lerner and Keltner, 2000). In the present study, participants in the anger condition, in the majority of specifications, evaluated the environmentally damaging entrepreneurial opportunities less favorably than participants in the Control condition. This pattern is consistent with, but does not directly demonstrate, an appraisal-based interpretation involving perceived responsibility or preventability because the study did not measure these appraisal processes. Future research should therefore test whether responsibility, controllability, or perceived preventability mediates the relationship between induced environmental anger and opportunity evaluation.

The non-significant finding of compassion requires a more cautious interpretation. The manipulation checks indicated that compassion was successfully induced, showing that the non-significant opportunity-evaluation findings cannot simply be attributed to a failed manipulation. However, compassion is generally an other-oriented emotion associated with sensitivity to suffering and tendencies toward helping or alleviating harm (Goetz et al., 2010; Lu and Schuldt, 2016). The dependent variable in the present study required participants to evaluate the attractiveness of a business opportunity rather than to decide whether to assist those affected by environmental harm. This potential mismatch between the target of the emotion and the judgment task is one possible explanation for why a statistically significant group difference was not detected. At the same time, successful relative induction does not establish that compassion was sufficiently intense to be relevant to the subsequent opportunity-evaluation task. Because neither the target nor different levels of compassion intensity were experimentally manipulated, these explanations remain tentative and should be examined directly in future research. The case-specific pattern for hope may indicate a potential boundary condition for the application of the Appraisal Tendency Framework in this judgment domain. Hope is associated with the perceived possibility that an undesirable situation can be improved or that a desired outcome remains attainable (Balen and Merluzzi, 2021; MacInnis and De Mello, 2005). One possibility is that the pattern associated with hope varies with participants’ perceptions of characteristics such as the controllability, severity, or reversibility of environmental harm. However, the air- and water-pollution scenarios used in this study were not designed to isolate these characteristics experimentally. It would therefore be inappropriate to conclude, for example, that the water-pollution scenario was perceived as more reversible or more amenable to corrective action than the air-pollution scenario. Instead, the present findings support only the more limited conclusion that the pattern for hope was context-dependent across the two scenarios. Future studies could directly manipulate or measure perceived severity, controllability, and reversibility to examine why the pattern associated with hope differs across evaluations of environmentally damaging entrepreneurial opportunities.

### 4.2 Positioning the findings

For environmental psychology, the principal contribution is that eco-emotions are examined here in the evaluation of environmentally damaging entrepreneurial opportunities, where environmental harm is an explicit feature of the opportunity being evaluated. The findings suggest that emotion-specific appraisal tendencies may carry over unevenly into this domain: anger showed the most consistent pattern, hope was context-dependent, and compassion showed no detectable difference in opportunity evaluations relative to the Control condition. Because the study did not directly measure the appraisal processes that might underlie these patterns, this evidence should be interpreted as identifying a domain and potential boundary conditions for the Appraisal Tendency Framework rather than as demonstrating a new appraisal mechanism.

More broadly, the results align with psychological research showing that emotions influence decision-making in an environmental context. New research provides a strong case for considering emotional intelligence (Marchetti et al., 2024) and discrete emotions (Paek and Hove, 2024; Vrselja et al., 2024) as relevant to understanding how individuals form their views on environmental issues. Additionally, Kesenheimer et al. (2025) report a stronger effect of negative valence emotions on pro-environmental decision-making than positive valence emotions. In the screened primary sample, the present results align with this literature in that the anger condition showed the most consistent pattern of lower evaluations of environmentally damaging entrepreneurial opportunities, whereas the pattern for hope was context-dependent and the compassion condition did not differ significantly from the Control condition.

More specifically, this study contributes to an established interdisciplinary literature stream of affect and entrepreneurial opportunities. The findings support the thesis that psychological factors, specifically emotions, are needed to explain certain dimensions of the entrepreneurial process (Frese and Gielnik, 2014). Specifically, the findings are best positioned alongside those of Grichnik et al. (2010), Ivanova et al. (2018), Ojha and Nandakumar (2025), Kollmann et al. (2017), and Foo (2011). Grichnik et al. (2010) demonstrated that induced positive and negative emotional states can have discrepant influences on opportunity evaluations and opportunity exploitations. Similarly, Ivanova et al. (2018) underscored the impact of anger, fear, and happiness on the desirability of opportunities. Likewise, emotions of shame (Ojha and Nandakumar, 2025) and fear of failure (Kollmann et al., 2017) are integral to the entrepreneurial opportunity framework. The most comparable studies to this one are those by Foo (2011) and Welpe et al. (2012). Foo (2011) established a relation between emotions and risk perceptions, reporting differences in risk assessments between anger- and hope-induced participants. This prior finding identifies risk perception as a potentially relevant process for future research. However, risk perception was not measured in the present study and therefore cannot be used to explain the current results. Furthermore, Welpe et al. (2012) identified anger as an emotion relevant to examining entrepreneurial opportunities, specifically the exploitation of envisaged business scenarios.

Moreover, this study’s findings add value to the environmental emotions literature. Discrete emotions related to the environment, often labeled as eco-emotions, have been the subject of scholarly scrutiny for a number of years. As more scientific work was carried out, the case for a multi-emotion-based framework for portraying environmental decision-making and stances became stronger (Cianconi et al., 2023; Marczak et al., 2023; Pihkala, 2022). Nevertheless, some emotions emerged as more prominent than others, such as anger. For instance, anger is a significant predictor of pro-environmental intentions and behaviors (Contreras et al., 2024), as well as a key determinant of participation in collective actions in response to environmental changes (Stanley et al., 2025). Within the screened primary sample, the largely consistent pattern of lower evaluations in the anger condition is compatible with the possibility that appraisal tendencies associated with environmental anger may extend to this evaluation context. This interpretation remains tentative because the appraisal processes were not measured directly and the pattern was not statistically robust to the full-sample sensitivity analysis after multiplicity adjustment. This study is also aligned with the literature on environmental compassion, where compassion is associated with helping behaviors rather than sanctioning responses (Garrido-Macías et al., 2023; Lu and Schuldt, 2016). This comparison suggests that the relevance of compassion may depend on the judgment context. The pattern for hope was less consistent: eco-hope was associated with lower evaluations in the Water case but not consistently across both entrepreneurial opportunities, aligning with studies that likewise report context-dependent or inconsistent relationships between hope and environmental outcomes. Ojala (2015) finds that the effect of hope on environmental engagement depends on how hope is conceived. Similarly, Chadwick (2015) found that the significance of hope for environmental interest is absent when the tested outcomes are behavioral intentions. Taken together, these comparisons situate the three emotions within distinct lines of environmental-emotion research while underscoring their context-dependent patterns.

From a practical perspective, the findings suggest that the emotional context surrounding environmentally damaging entrepreneurial opportunities may be relevant to how such opportunities are evaluated. For entrepreneurs, policymakers, and other stakeholders, the results highlight the potential importance of considering emotional responses when seeking to understand evaluations of projects involving explicit environmental harm. In particular, the pattern observed for anger indicates an association between the anger condition and less favorable evaluations in the screened sample, but it does not establish why stakeholders respond to real-world projects. However, these findings should not be interpreted as evidence that inducing a particular emotion will produce a desired behavioral response. Because the study did not test specific communication strategies or real-world entrepreneurial decisions, these practical implications should be regarded as preliminary and as a basis for further field-based research rather than as prescriptive recommendations.

### 4.3 Study limitations and future research

The first limitation concerns the external and ecological validity associated with the sample composition. The participants were undergraduate students rather than practicing entrepreneurs or investors. Student samples can be appropriate for experimentally testing theoretically specified relationships in entrepreneurship research (Hsu et al., 2017), particularly when the research question concerns opportunity evaluation. Nevertheless, this methodological justification should not be interpreted as evidence that students’ judgments are equivalent to real entrepreneurial decisions. Unlike practicing entrepreneurs or investors, participants in this study did not face financial risk, resource commitments, accountability to stakeholders, or actual implementation consequences. Accordingly, the findings provide evidence about the evaluation of hypothetical entrepreneurial opportunities under experimental conditions, but their generalizability to real-world entrepreneurial behavior remains to be established. Comparable studies linking emotions and entrepreneurial opportunities have also used student samples (Ivanova et al., 2018; Kollmann et al., 2017; Welpe et al., 2012), which supports the use of the design for theory testing but does not remove this external-validity limitation.

Second, participants were presented with concise descriptions of entrepreneurial opportunities. Although concise scenarios facilitate experimental control by limiting extraneous information, they necessarily simplify the entrepreneurial decision context. Real-world opportunity evaluations would typically include information on expected revenues and costs, financing requirements, regulatory constraints, implementation risks, community and stakeholder responses, and the magnitude and reversibility of environmental consequences. The absence of such information may have influenced participants’ judgments and limits the ecological validity of the experimental task (Ivanova et al., 2018).

Third, the study was conducted with participants from one country, Croatia. This choice limits the applicability of the results to other cultural contexts because cultures may differ in emotion regulation, affect valuation, and the intensity or expression of emotional experience (Chen et al., 2025; Tsai et al., 2025). Consequently, neither the magnitude nor the pattern of the observed emotion-evaluation relationships should automatically be generalized beyond the Croatian context. Cross-cultural replications would be particularly valuable for determining whether the patterns observed here are stable across societies characterized by different environmental norms, affective cultures, and entrepreneurial environments.

Fourth, effect sizes were generally small, and the two largest estimates approached but did not reach Cohen's (1988) conventional medium benchmark. This pattern potentially limits the real-world applicability of the study. Two things are worth mentioning on this point, both underscored by Götz et al. (2022). First, it is preferable to contextualize the magnitude of effect sizes over interpretations made in isolation. Thus, whether the reported effect sizes are practically consequential cannot be fully determined from the present study, because no established benchmark for a minimally meaningful difference in opportunity evaluation is available. Second, psychology, given the multiplicity of studied concepts, is unlikely to find one or two concepts which explain shifts in desired outcomes. Rather, the more plausible scenario is that psychology develops as a cumulative science, where many concepts, with even small effect sizes, help in explaining desired outcomes. Hence, while large effects are still favored over small ones in psychology (Primbs et al., 2023), they do not fully jeopardize the practical relevance of this study. Another potential point worth addressing on the topic of effect sizes is the notion of large samples being the main drivers of small effect sizes. While possible, this statistical issue is not the most probable cause of small effect sizes in this study since sample sizes need to be much larger and the effect sizes need to be much smaller than those of this study for this statistical feature to occur (Combs, 2010).

Fifth, the non-replicability of the findings using a full sample might raise concerns about the models’ robustness. Yet, when examining the methodological literature, such an occurrence is not surprising. Oppenheimer et al. (2009) and Maniaci and Rogge (2014) highlight that unengaged participants can introduce statistical issues, such as an increase in measurement error and reduced statistical power. These and other occurrences can cause a shift from statistically significant to statistically non-significant estimates when final samples are compared to full samples (Mathur, 2025). Although samples containing only engaged participants can introduce selection bias (Montgomery et al., 2018; Mathur, 2025), the screened sample is retained as the primary inferential sample because the engagement criteria were intended to reduce measurement error associated with inattentive responding, while the full sample is reported as a sensitivity analysis. The central inferences should therefore be understood as conditional on the screening procedure. Several mentioned limitations help to identify priorities for future research. Subsequent studies could replicate the observed emotion-opportunity evaluation patterns with practicing entrepreneurs, in geographically diverse samples, or using more detailed case studies. The first two recommendations should be more easily implementable than the third, since devising entrepreneurial opportunity scenarios of any kind requires methodological rigor and prior testing (Aguinis and Bradley, 2014). Any new opportunity scenarios should therefore be developed and pretested carefully.

A second line of future research could incorporate a broader range of emotions into empirical models. This study examined only anger, hope, and compassion. Portocarrero et al. (2025), in their expansive review of the entrepreneurial affect literature, identified many other emotions relevant to the entrepreneurial process, including admiration, disgust, pride, regret, and shame. These emotions have a prior empirical basis in the entrepreneurship domain, potentially justifying their use in the context of environmentally damaging entrepreneurial opportunities. However, as with the suggestion to provide alternative opportunity scenarios, much work needs to be done before testing these models. Zaremba et al. (2024) conducted a detailed study to elicit environmentally specific emotions, which was used in this study. This research agenda could proceed in two stages. First, studies could develop and validate environmental scenarios for a wider range of emotions, following Zaremba et al. (2024). Second, the validated scenarios could be incorporated into empirical tests of entrepreneurial opportunity evaluation.

Lastly, future research could add more nuance to the experienced emotions, as allowed by the Appraisal Tendency Framework. Indeed, appraisal tendencies are said to vary by intensity and by duration (Lerner et al., 2015; Lerner and Keltner, 2000). Although participants reported their experienced emotions, the design did not experimentally manipulate emotion intensity or duration. Future research could manipulate or measure these dimensions more directly and examine whether they condition evaluations of environmentally damaging entrepreneurial opportunities.

## 5 Conclusion

This study examined evaluations of environmentally damaging entrepreneurial opportunities across experimental conditions inducing hope, compassion, anger, or a control state. Within the experimental task, participants in the anger condition almost consistently assigned lower evaluations to these environmentally damaging entrepreneurial opportunities. The pattern for hope was context-dependent, appearing for the water-pollution scenario but not consistently across both scenarios, whereas the compassion condition did not differ significantly from the Control condition in opportunity evaluation. These findings support an emotion-specific view of entrepreneurial opportunity evaluation when environmental harm is an explicit feature of the opportunity and suggest that appraisal tendencies associated with environmentally relevant emotions may extend to this judgment context. However, the results concern hypothetical judgments made by Croatian undergraduate students rather than actual entrepreneurial behavior. They suggest only that emotional context may be relevant to evaluations of such opportunities; they do not explain why stakeholders support or oppose specific real-world projects. The practical implications should therefore be considered preliminary. The study did not test communication interventions or real-world decisions by entrepreneurs, investors, policymakers, or other stakeholders, and the findings should not be used to prescribe specific managerial or policy interventions without further validation.

## Data Availability

The raw data supporting the conclusions of this article will be made available by the authors, without undue reservation.
