# Supplementary material for: The impact of emotions on the evaluation of environmentally damaging entrepreneurial opportunities: an experimental study
Source: Front Psychol. 2026 Sep 16;17:1893664. doi: 10.3389/fpsyg.2026.1893664 (PMC13623600; doi:10.3389/fpsyg.2026.1893664)
Supplement: Supplementary file 1 [file Supplementary_file_1.DOCX]

Supplementary Material

**The impact of emotions on the evaluation of environmentally damaging entrepreneurial opportunities: An experimental study**

| **Survey item** | **Source** |
| --- | --- |
| **Hope story 1:** Thanks to the prompt and coordinated action of governments, it was possible to ban the use of substances that deplete the ozone layer in the atmosphere. After some time, it was observed that the ozone layer was recovering. This shows that humanity is capable of dealing with environmental disasters effectively.  **Hope story 2:** Emily watched an inspiring lecture on possible solutions to the problem of climate change. She then published a post about it on social media and sent a recording of it to her friends. Emily always thought that hardly anyone among her acquaintances was interested in the climate, but it turns out that the post has been viewed and commented on by hundreds of people.  **Hope story 3:** Esther's daughter told her mother that the management of her school was considering the installation of solar panels. Hearing that, Esther asked other members of the Parents Council to support the director's initiative. One parent is an accountant, and he volunteered to help process the purchase of panels for the school, free of charge.  **Hope story 4:** Isabella used to commute to work by car. When she switched to a bicycle, she had to get used to the new way of transportation. Today Isabella would not want to switch back to the car – she likes the opportunity to exercise in | Zaremba et al. [21] |
| the morning, while avoiding traffic jams and at the same time making a contribution to climate protection. She has also convinced a few colleagues to switch to the bike.  **Hope story 5:** Elliah is disappointed with the decision of the local authorities, who, due to the lack of funds, abandoned the plan to remodel the square paved with concrete and plant some trees and shrubs. Elliah organised a group of friends who, acting on their own, removed the paving slabs that covered most of the square. A gardener friend of his donated plants, which they planted in place of the concrete.  **Hope story 6:** Amara is an engineer and she specialises in fire safety. She has invented a special coating for buildings to protect them from fires, which are increasingly common. What is more, the coating is made of industrial waste, which would normally end up in a landfill.  **Hope story 7:** Yoshida has discovered a new species of bacteria. The bacteria are able to break down plastic that would otherwise be deposited in landfills. Yoshida's discovery gained so much publicity that he was awarded another research grant. There are reasons to hope that his solution can be implemented on a large scale. |  |
| **Compassion 1:** Hanna and Jerry, who are now retired, spent most of their lives in a house on the edge of a forest. This year, they had to abandon it, after a wildfire had destroyed the house and all their belongings. The fire was most likely due to excessive heat caused by climate change.  **Compassion 2:** Halima's family suffered a typhoon that devastated her hometown on the coast. Destroyed infrastructure, flooded homes and difficulties in bringing help to the wounded and victims are the various consequences of humanitarian disasters caused by climate change.  **Compassion 3:** Amber comes from a family that has been farming the land for generations. Due to climate change, the soil has become dry and barren. Amber has no choice but to sell the family farm for very little money and look for a different way of life. “This place is more than just a source of income to me.”, says Amber with tears in her eyes.  **Compassion 4:** Jacob and Kayla suffer greatly after the last move. Due to a disaster at a nearby coal mine, the authorities relocated the elderly couple from their family home. Jacob feels like a stranger in the new place, and Kayla keeps thinking about how good their life used to be.  **Compassion 5:** Betty can't go outside because of the heatwave. She is old and weak, and such extreme temperatures impact her health. She feels lonely and trapped in her apartment. Sitting in front of the fan all day, she wonders how long she will have to live this way.  **Compassion 6:** Scott and his family went on vacation to Croatia, to a place he used to visit in the past. He recognized familiar places, but noticed that there were fewer people there. Due to drought, olives, which local farmers used to press, are in short supply. Deprived of this source of income, they migrated to cities with their entire families, leaving their homes behind. Scott is sorry to see the abandoned and decaying farms.  **Compassion 7:** Tina switches to another TV channel, as she can no longer bear to look at the images from Eritrea. The prolonged drought leads to hunger, which kills little children. While falling asleep, Tina still can't get over the thought of innocent people suffering from the effects of climate change. They did nothing to contribute to it, after all. | Zaremba et al. [21] |
| **Anger 1:** A certain politician downplayed reports about the threat of drought caused by climate change. When the town suddenly ran out of water, he sneaked off to his lakeside summer residence to avoid journalists.  **Anger 2:** A corporation commissioned a report on its environmental impact. As the report turned out to embarrass the corporation, the management decided to conceal it. Employees were forbidden to disclose any information on this subject.  **Anger 3:** Once a week Frank buys groceries at a large supermarket. Each time he asks the cashier for new plastic shopping bags. When he brings his shopping home, he throws the bags away without a second thought.  **Anger 4:** Eila's uncle claims that climate change is a fabrication and an excuse to increase energy prices. When Eila tries to convince him that the situation is serious, her uncle criticizes her for wasting too much time reading nonsense on the Internet and allowing herself to be manipulated by social media.  **Anger 5:** A certain species of pine is dying out in local forests as a result of climate change. Instead of planting trees better adapted to a dry, hot climate, the authorities keep planting the same species, because they benefit from a contract with the supplier of seedlings.  **Anger 6:** August is a senator and his wife works at a state-owned company responsible for heavy environmental pollution. August blocks financial support for climate action to protect his wife's financial interests.  **Anger 7:** Lisha is very rich and likes to change her wardrobe frequently. Ever since she has discovered a website with very cheap clothes, she orders 20-30 items a week. Some turn out to be a poor fit, so she throws them straight into the garbage bin. Still, Lisha does not consider this wasteful, because the clothes were ridiculously cheap. | Zaremba et al. [21] |
| **Control 1:** John entered the conference room and sat down on a chair. Only then did he look around and think, “If the front row seats have not all been taken yet, I'll switch.” He got up and moved closer to the screen at the front of the room.  **Control 2:** Anne hasn't changed the recording on her voicemail for a long time. It occurred to her that she might make the greeting more professional. She recorded a new message and listened to it. She was pleased with what it sounded like, so she saved it and put the phone down.  **Control 3:** Monica entered the room, opened the wardrobe and bent down to reach into one of the lower shelves. She took out her brown pants and put them on. Then she opened a drawer in her dresser and took out a belt. She passed it through the belt loops on her pants and fastened the buckle.  **Control 4:** Milo is driving a car with his wife and children in passenger seats. It is quiet – the children are sleeping in the back seats, and his wife calmly watches the road. A slight drizzle has just started. Milo turns on the wipers to clean the windshield and improve visibility.  **Control 5:** Having watched the last episode of his favourite series, Olivier asked his friend if he could recommend another one. Olivier got several recommendations, read the reviews and chose one title. The first episode turned out to be very interesting, so he intends to keep watching it.  **Control 6:** Adam is expecting guests. He entered the bathroom to check if there was soap in the dispenser and noticed that there was no towel. “Do I have any clean towels?” he thought as he left the bathroom to look for a towel in the closet. He took a small beige towel and returned to the bathroom to hang it on the hook next to the sink.  **Control 7:** George decided to take a break from work and have some lunch. He usually eats a bun, but this time he only had some bread at home. He cut two slices, took the rest of the ingredients out of the refrigerator, and made sandwiches. He took the plate with the sandwiches to his desk and ate them there as he browsed a news website. | Zaremba et al. [21] |
| **Case study air:** Ge Wang, Yang Liu’s college friend, has been interested in refrigeration technology, and has been trying to create a refrigerant with a better cost-effectiveness. Lately, Ge has created one that has better performance but lower cost than those commonly found in the market. Ge soon has patented this new product. Because the refrigerant project has been greatly supported by Yang, Ge is interested in industrializing the newly created refrigerant with Yang. Ge proposes to establish a new factory to produce approximately 10,000 tons of the refrigerant in a city with good transportation service. Discovering Ge’s innovation, some potential investors are interested in financing Ge and Yang’s business project. What is more, a local business park is also interested in this project and has invited Ge and Yang to register their firm in the business park. It also promises good financial and tax support. However, in a series of tests of the new refrigerant for environmental influences, it is found that this project may have negative impacts on the local atmospheric balance.  1. Chemistry test suggests that when heated, the new refrigerant will generate a type of greenhouse gasses that is commonly known to cause temperature rise. The test suggests that the greenhouse gas under concern is 250 times more effective than carbon dioxide in storing heat.  2. A local meteorological bureau estimates that greenhouse gas that would be generated by Ge and Yang’s factory can lead to a 5% increase in temperature annually in areas affected.  3. A study based on the factory’s five-year production plan further estimates that greenhouse gas generated during the period would significantly increase local temperature, to an extent that is equivalent to 10% of heat rise caused by carbon dioxide during the same period in the area. | Qin et al. [20] |
| **Case study water:** City L is famous for its beautiful lake, fairy lake. Located at a plateau, the lake is an excellent spot for photographing of the sunrise and sunset. However, the lake is at a long distance from the city L, and no convenient transportation service is available for travelers to reach the lake. In addition to the transportation inconvenience is the poor infrastructure surrounding the lake, such as accommodation buildings. Because of these reasons, the fairy lake has a very limited capacity to host travelers and photographers. Yang sees an opportunity of developing the fairy lake into a popular tourist site. Having done some research, Yang found a suitable place near the lake for building a 50-room resort hotel. Tourists can stay at the hotel in order to catch the moment of  sunrise and sunset. Yang has contacted several venture capitalists about this project, and has received favorable opinions from them. What is more, the local government of city L is very supportive for Yang’s project because of potential economic growth brought by it. It is estimated that after the hotel starts to operate, it will contribute more than one million RMB to the government’s annual tax revenue. Yang’s hotel will also create about 150 new jobs for the local city. One of Yang’s concerns, when selecting the location for the hotel, is potential pollution brought by the hotel sewerage system. He has discovered that the lake is connected to a stream of another river, which would be used to connect to the hotel’s sewerage system, reducing the possibility of polluting the lake directly. Despite this discovery, Yang still paid an environmental impact assessment (EIA) agency for an exclusive test. According to the EIA agency’s test report, the hotel may have negative impacts on surrounding areas of the lake. The content of the report are as follows:  1. Domestic sewage created from the hotel can lead to water eutrophication, a kind of water pollution. The test estimates that domestic sewage created by each hotel room is 50% greater than that by a common household.  2. The ecological environment of the plateau is too delicate to self-purify water waste created by the hotel. This can upset the ecological balance in the local area.  3. Water eutrophication problem created by the hotel’s domestic sewage can further break the ecological balance in a river near the fairy lake. The water eutrophication will lead to demise of certain rare type of fish and water plants. | Qin et al. [20] |
| **Case study neutral:** Mr. Tan is a successful manager with four years of experience at a multi–national corporation (MNC). Before that he worked in a medium sized local company for five years. The idea of being his own boss, taking calculated risks, and making a fortune all appeal to him. Hence he is thinking of starting his own business. He has an idea for a new business and decides to ask around to see if it is a good idea. He has some very positive feedback from some potential customers and some associates who know the industry well. Mr. Tan does not have the resources to do an in–depth market research to find out whether the business is going to work and published data are too general to be useful. However he feels that there is money to be made based on the positive feedback from potential customers and his associates. He is enthusiastic about starting the business even though he has no experience in this industry or starting his own business. There are a few MNCs in the same industry but they have not targeted the market segment that Mr. Tan is aiming for. He feels that the MNCs are likely to move into the market as long as the new business is successful and he will not be able to fend off this major threat. He is unsure whether the market is still growing or matured. If the market has reached maturity, it is likely for a new business to be squeezed out of the market. If the market is still growing, the new business will be able to survive the entry of MNCs into this market segment. He finds out that there are only a few small businesses that are still surviving in the industry. Mr. Tan estimates he will need at least S$150,000 to finance the new business. As he has only S$40,000 in savings, he has to borrow from the bank or find partners to get the rest of the investment funds needed. | Keh et al. [23] |
| **Opportunity Evaluation:**  1. I consider this business an opportunity  2. This business is worth considering  3. This business is feasible given the situation | Keh et al. [23] |
| **Nature Disengagement Scale**  1. It is okay to sacrifice the natural environment for the economic development.  2. Polluting the nature environment is okay as long as you can restore the environment later.  3. Considering that other people are destroying the natural environment, it’s hardly a sin to litter the rubbish casually.  4. People shouldn’t be held accountable for doing things that harm the natural environment when they were just doing what an authority figure told them to do.  5. People can’t be blamed for doing things that are bad for the natural environment when all their friends are doing it too.  6. Doing things that harm the natural environment for the present is no big deal.  7. The natural environment can be harmed because it can restore itself.  8. The natural environment that was damaged was holding back human survival and advancement. | Qin et al. [20] |
| **Demographic**  1. What is your gender?  2. What is your age?  3. Do you have prior work experience?  4. Is anyone in your family an entrepreneur? | Standard items |
| **Emotion check**  To what extent do you fell:  1. anger  2. hope  3. compassion  4. guilt  5. anxious | Standard items |
| **Hope attention check:** What can Yoshida's discovery decompose?  a) Paper  b) Plastic  c) Glass  **Compassion attention check:** What is the cause of famine in Eritrea?  a) Prolonged drought  b) State of war  c) Political crisis  **Anger attention check:** What does Lisha do with the clothes she orders online that don't fit her?  a) Throw them in the trash  b) Donate them to the needy  c) Returns them  **Control attention check:** What did George prepare and eat?  a) Eggs  b) Pancakes  c) Sandwiches  **Air attention check:** What did Ge patent?  a) Refrigerant  b) Solar panels  c) New type of batteries  **Water attention check:** What does Yang want to build near the lake?  a) Golf courses  b) Hotels  c) Theme parks | Author-devised checks |
